# Preoperative Botulinum Toxin and Progressive Pneumoperitoneum in Loss of Domain Hernias—Our First 100 Cases

**DOI:** 10.3389/fsurg.2020.00003

**Published:** 2020-02-28

**Authors:** José Bueno-Lledó, Omar Carreño-Saenz, Antonio Torregrosa-Gallud, Salvador Pous-Serrano

**Affiliations:** Surgical Unit of Abdominall Wall, Department of Digestive Surgery, “La Fe” Universitary Hospital, University of Valencia, Valencia, Spain

**Keywords:** incisional hernia, ventral hernia, large incisional hernia, preoperative progressive pneumoperitoneum, botulinum toxin

## Abstract

**Objectives:** Preoperative botulinum toxin type A (BT) and progressive pneumoperitoneum (PPP) are useful tools in the preparation of patients with loss of domain hernias (LODH). The purpose of our retrospective study is to report our experience in the treatment of 100 consecutive patients with LODH, with the combined use of these techniques.

**Methods:** Of the 753 patients operated on for ventral incisional hernia between June 2010 and December 2018 in our hospital, 100 patients with LODH were analyzed retrospectively. Diameters of abdominal cavity and hernia sac, and volumes of incisional hernia (VIH) and abdominal cavity (VAC) were calculated from CT scan, based on the index of Tanaka.

**Results:** The median insufflated volume of air for PPP was 8,600 ± 4,200 cc (4,500–15,250). BT administration time was 38.2 days (33–48). A significant average reduction of 15% of the VIH/VAC ratio was observed on CT scan after the combination of PPP and BT (*p* = 0.001). Anterior component separation (CST) and transversus abdominis release (TAR) were the most frequent repair techniques. Complete fascial closure was possible in 97%, and mesh bridging was needed in three cases. In postoperative follow-up of 34.5 months (11–62), we reported eight cases of hernia recurrence (8%).

**Conclusion:** PPP and BT are useful tools in the treatment of LODH. These techniques significantly reduce the VIH/VAC ratio, allowing the reduction of the hernia content into the abdominal cavity, which represents a key factor in the management of these hernias.

## Introduction

Loss of domain hernia (LODH) represents a challenge for the abdominal wall surgeon. Continuous persistence of a significant part of the intestinal package in the hernia sac may cause respiratory disorders, physiological alterations, and lifestyle changes in these patients (1).

Preoperative tools like progressive pneumoperitoneum (PPP) or botulinum toxin type A (BT) have been reported as useful in the preparation of patients with this type of hernias (2–4). BT infiltration on the abdominal wall has been considered a chemical component separation because of its lack of adverse effects and its temporary action. It also presents the advantage of continued effect in the late postoperative period, ~6 months during which the operated abdomen adapts to these changes (5). On the other hand, PPP has brought a significant change in the surgical approach to LODH, reducing complications such as abdominal compartment syndrome (ACS) and restrictive pulmonary disease (RPD) (6). Although it is not universally used in most hospitals, specialized groups have reported good results with this procedure, with acceptable risks (7, 8).

Both procedures are complementary to the hernia repair, using no-strain mesh techniques that support patient's integral management. The purpose of this observational retrospective study was to report our experience in the management of our 100 first consecutive cases of LODH, with the combined use of BT and PPP.

## Methods

**Figure 1:**
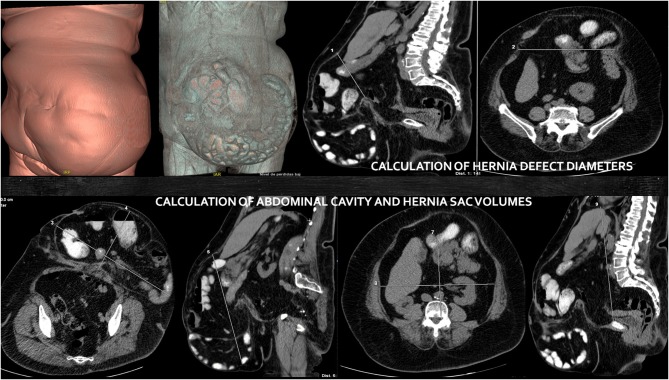
Volumetric CT images before botulinum toxin and progressive pneumoperitoneum application. Calculation of hernia sac and abdominal cavity volumes and hernia defect diameters.

Of the 753 patients operated on ventral incisional hernia between June 2010 and December 2018 in our hospital, we analyzed retrospectively 100 consecutive patients with LODH, by prospective collected data. Diameters of the abdominal cavity and the hernia sac, as well as the volumes of the incisional hernia (VIH) and abdominal cavity (VAC) were calculated from a multidetector CT scan, based on the index of Tanaka (9) (Figure 1).

**Figure 2:**
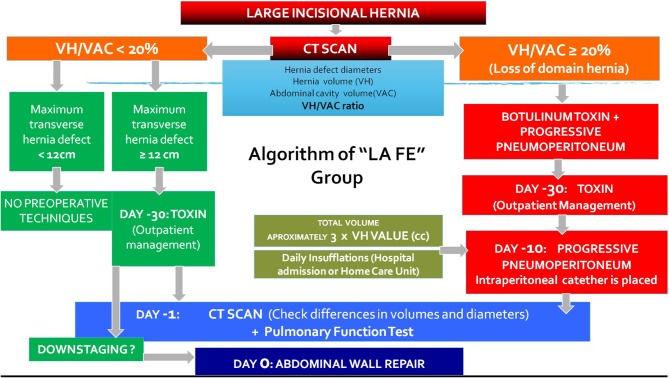
Algorithm of combination of botulinum toxin and progressive pneumoperitoneum in the treatment of large incisional hernias applied in our hospital.

Considering these measurements and physical examination, our group created an algorithm that was approved by our hospital's ethics committee (Figure 2). A VIH/VAC ratio was calculated. If this ratio was <20%, the procedure of choice depended on the location and size of the hernia (CST, TAR, Rives-Stoppa repair, etc.), and this is a factor for not being a candidate for our study. However, if the VIH/VAC ratio was higher or equal to 20%, these patients were included in the protocol of preoperative techniques, based on reported results and indications of PPP by other groups (8, 9). Preanesthetic evaluation and pulmonary function tests were carried out in all patients, and their informed consent for the preoperative techniques was obtained. In the preoperative assessment, we emphasized on the importance of smoking abstinence and weight loss in obese patients.

**Figure 3:**
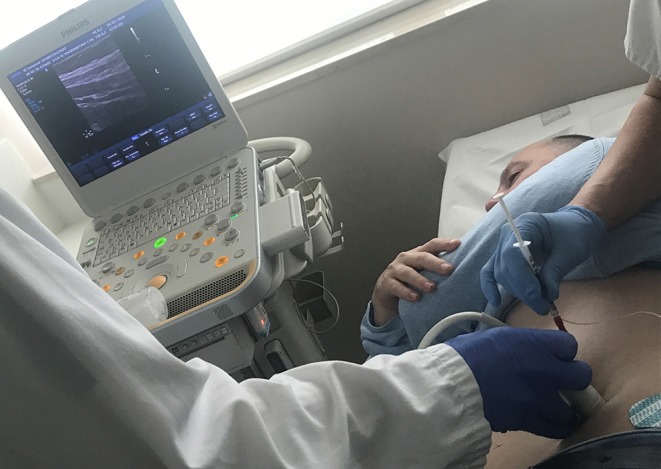
Botulinum toxin application under ultrasonographic and electromyographic guidance on the lateral abdominal wall.

BT injections were performed as an outpatient procedure, 4 weeks prior to their planned operation, according to a previous description of the technique (3). The location of the points for injection was guided by ultrasound, visualizing the three lateral muscle layers to be infiltrated (external oblique, internal oblique, and transversus abdominis). The patient was placed in a lateral position, following the Ibarra–Hurtado model. A 5-ml injection of Dysport (50 U/5 ml; Ipsen, Boulogne-Billancourt, France) was administered at each point bilaterally, for a total of 500 U, under ultrasound vision into each of the three muscle bellies of the lateral muscles. In our case, EMG guidance with a monopolar electrode of 75 mm complimented the ultrasound location, confirming whether the muscle where we applied BT was denervated or fibrotic, or modifying the injection point to another muscular area, ensuring its effectiveness (Figure 3).

**Figure 4:**
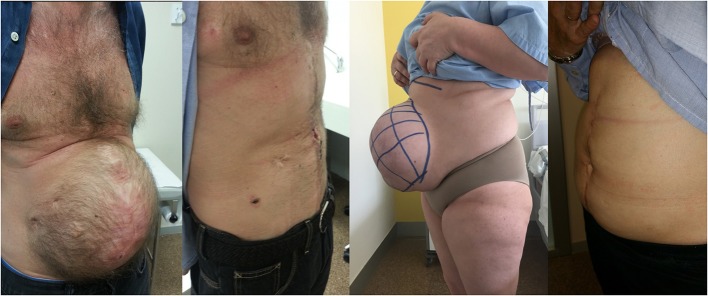
Comparation of pictures of patients after combined administration of botulinum toxin and progressive pneumoperitoneum and after following hernia repair.

To apply PPP, an intraperitoneal double-lumen catheter was placed preferably in the upper left quadrant, as has been previously described (10). Approximately, the total volume of insufflation was three times the volume (cc) of VIH in CT findings. This volume was introduced for 1–2 weeks for progressive adaptation: an initial insufflation between 500 and 1,000 cc of ambient air was carried out, monitoring the patient and asking him about the appearance of symptoms such as abdominal pain or dyspnea. Subsequently, 500–1,000 cc of air to complete the scheduled daily volume was administered. A prophylactic enoxaparin injection was administered daily during insufflation (Figure 4). Pneumoperitoneum was maintained until the day before surgery, where diameters and new volumes were evaluated again by CT scan.

All patients underwent abdominal repair under general anesthesia and abdominal bandage during the postoperative period. Hernia repair was performed trying fascial defect closure with mesh placement. All the surgical repairs were performed by five surgeons dedicated to hernia repair and members of Unit of the Abdominal Wall Surgery.

Most of the repairs were anterior component separation (CST), using a modified technique as we have previously described (11). After the rectus muscles were re-approximated in the midline, a polypropylene or PVDF mesh was placed onlay and was anchored in some cases with non-absorbable tackers or sutures (Prolene) to the costal margin, anterior iliac spine, and pubis, located between the internal and external muscles. Finally, a myoplasty to fix the flap of the external oblique muscle to the mesh was carried out. In the rest of the cases, transversus abdominis release (TAR) or Rives–Stoppa repair was performed. A synthetic mesh was used to cover the defect within the retromuscular space, aiming at the generous reinforcement of the entire visceral sac. In the case of TAR, the mesh could be placed laterally into the retroperitoneum next to the lateral edge of the psoas muscle.

Removal of excess skin and subcutaneous tissue was performed prior to closure of the incision, and two to three closed suction drains were used; all drains were removed when drainage was <30–40 cc/24 h. A scheduled recovery in the Intensive Care Unit (ICU) for the initial postoperative period in some cases was performed. Postoperative follow-up was carried out at 15 days, 4, 8, and 12 months with annual controls later.

Demographic data, American Society of Anaesthesiologists (ASA) status, history of active smoking, chronic obstructive pulmonary disease (COPD), clinical and surgical history, hernia location, preoperative technique, measurements of abdominal volumes (VAC, VIH) and diameters (hernia and abdominal cavity) on CT scan, VIH/VAC ratio, type of repair, type of prosthesis, operative time, length of hospital stay, postoperative follow-up, and hernia recurrence rate were recorded. PPP, BT, and postoperative complications by the Clavien–Dindo classification (12) were recorded. Hernia recurrence was determined by clinical examination (a palpable mass at the site of the previous hernia repair) plus confirmation by abdominal CT scan.

Descriptive statistics including means and standard deviations for continuous variables were used. Frequency tables for the recorded variables and corresponding dispersion measures were calculated. Univariate analysis was performed using “t-Student” or Wilcoxon test to explore quantitative variables and “Chi square” (or Fisher test) if they were dichotomous. A value *P* < 0.05 was considered to be statistically significant. Statistical analysis was performed using SPSS (version 21.0).

## Results

One hundred consecutive patients with LODH who had undergone elective repair between June 2010 and December 2018 were analyzed. A combination of PPP and BT was performed in all patients. Demographic and preoperative variables of the patients are shown in Table 1.

**Table 1:** Demographics and characteristics of the patients with LODH.

**Variables**	**BT** **+** **PPP** ***n*** **=** **100**	
Mean age (range)	59.4 (33–81)	
**Gender (%)**		
Male	41 (44.2)	
Female	59 (55.8)	
First abdominal surgery (%)	Umbilical hernia	19 (19)
	Colectomy	17 (17)
	Cesarea	15 (15)
	Cholecystectomy	11 (11)
	Trocar hernia	9 (9)
	Renal transplantation	9 (9)
	Hepatic transplantation	8 (8)
	AAA	6 (6)
	Gastric bypass	6 (6)
Time of evolution since the first surgery in years (range)	8.5 (2.2–16)	
**Active smoking**		
Yes	11 (11)	
No	89 (89)	
**ASA score (%)**		
Grade I	17 (17)	
Grade II	54 (54)	
Grade III	29 (29)	
OBESITY (BMI >30) (%)	19 (19)	
**Hernia defect location**		
Midline	82 (82)	
Lateral	18 (18)	
Median operative time in min (range)	223 (110–379)	
Abdominal wall repair technique (%)	Anterior CST	57 (57)
	Level	40
	Level 1+2	14
	Level 1+2+bridging	3
	TAR	32 (32)
	Rives-Stoppa	11 (11)
Technique associated with prosthetic repair (%)	Abdominoplasty	11 (11)
	Colectomy	4 (4)
	Intestinal resection	5 (5)
	Cholecystectomy	4 (4)
	Colostomy	3 (3)
Reoperation during hospitalization	8 (wound infection, dehiscence and necrosis) 1 CST: bridging with PTFE mesh	
Length of postoperative hospital stay in days (range)	9.4 (5–22)	
Average postoperative follow-up in months (range)	34.5 (12–62)	
Recurrence (%)	8 (8)	

*PPP, progressive preoperative pneumoperitoneum; BT, botulinum toxin A; SD, standard deviation; AAA, abdominal aortic aneurysm; CST, component separation technique; PVDF, polyvinylidene fluoride; ACS, abdominal compartment syndrome*.

Average time of BT administration was 38.2 days (33–48 days). Median insufflated volume of air for PPP was 8,800 cc (4,500–19,500), with an average of 1,000 cc (500–1,500) introduced during each session. Median insufflation time was 12.3 days (9–22 days). A comparison of peritoneal volumes and hernia defect diameter before and after preoperative techniques is shown in Table 2. A significant average reduction of 15% of the VIH/VAC ratio was observed on CT scan after the combination of PPP and BT (*p* = 0.001).

**Table 2:** Peritoneal volumes and hernia defect diameters before and after preoperative techniques.

	**Before PPP+BT** **(range)**	**After PPP+BT** **(range)**	***P***
VIH (cc)	1517 (615–2834)	2019 (535–3822)	0.004
VAC (cc)	9322 (4451–13001)	10910 (7780–14350)	0.011
VIH/VAC ratio (%)	29.1 (21.5–36)	14.1 (3.5–25)	0.001
Median transverse diameter of hernia defect (cms)	16.1 (12–22)	15.1 (12–22)	0.455

*PPP, progressive preoperative pneumoperitoneum; BT, botulinum toxin A; VIH, incisional hernia volume; VAC, abdominal cavity volume*.

No complications related to the administration of BT were reported (Table 3). In 10 patients, applying BT in the transverse muscle was not feasible due to lack of muscle layer in the injection site. Complications associated to PPP were 17%. In three cases, there were accidental punction related to catheter placement: one perforation of the small bowel and two intraabdominal hematomas, which were detected during the procedure, and laparotomy was not needed.

**Table 3:** Complications associated to the PPP and hernia repair according to the Clavien-Dindo classification.

**Complications** **(grade)**	**Associated to PPP** ***N* (%)**	**Treatment PPP** **complications**	**Associated to repair** ***N* (%)**	**Treatment of postoperative surgical complications** **(*N*)**
**Total (%)**	**17 (17)**		**27 (27)**	
Grade I	Shoulder pain 6 (6) Abdominal pain 5 (5) Subcutaneous emphysema 3 (3) Retroneumoperitoneum 1 (1)	Analgesics Analgesics Stop insufflations for 3-4 days Replacement catheter of insufflations	Seroma 10 (10) Wound infection 5 (5) Adynamic ileus 5 (5) Urinary retention 2 (2) Hematoma 3 (3)	No need of surgery Surgical debridement No need of surgery Parenteral nutrition 3-5 days Bladder catheter No need of surgery (2) Surgical debridement (1)
Grade III	Intraabdominal hematoma 2 (2)	No need of surgery Ultrasonographic control	Wound necrosis 4 (4) Mesh infection 3 (3)	Surgical debridement (2) Surgical debridement and NPT (2) No need of explantation (1) Mesh removal and TAR (2)
Grade IV	Intestinal perforation 1 (1)	No need of surgery Abdominal CT control	ARDS 2 (2) ACS 2 (2)	Ventilatory support in ICU Need of disassembly of the previous abdominal wall closure: bridging with PTFE mesh

There were no complications during the administration of BT.

*PPP, progressive preoperative pneumoperitoneum; ARDS, respiratory distress syndrome; ACS, abdominal compartment syndrome; TAR, transversus abdominis release; NPT, negative pressure therapy; ICU, intensive care Unit; CT, abdominal tomography; PTFE, polytetrafluoroethylene*.

Several reconstructive techniques were carried out. The CST and TAR were the most frequent hernia repairs. Median operative time was 223 min (110–379 min). Complete fascial closure was possible in 97%, and mesh bridging was needed in three cases. During adhesiolysis of the hernia sac, five bowel resections due to intestinal perforation were performed, without complications in postoperative course. Complications associated to surgical technique were 27%, most of them grade I, according to the Clavien–Dindo classification. Two patients with preoperative comorbidities suffered severe complications such as ACS (bladder pressure of 31 mmHg) associated with acute respiratory distress syndrome (ARDS), needing disassembly of the previous abdominal wall closure in one of them, performing partial fascial closure of the edges, and bridging with PTFE mesh. Median length of hospital stay was 9.4 days (5–22 days).

In the postoperative follow-up at 34.5 months (11–62 months), eight cases of hernia recurrence were reported, and 31% occurred within the first year following repair. Recurrence location was suprapubic in three patients (37.5%), lateral to the prosthesis in one patient (12.5%), and subxiphoid in four cases (50%). Especially, repair failed in three patients who developed deep wound infection, in two patients with a previous mesh bridging, and in another case complicated by a mesh infection after the main reconstruction. In total, six patients (75%) needed a posterior component separation with TAR to solve the recurrence, but two patients (25%) refused an additional repair surgery due to advanced age or an absence of symptoms. Finally, mesh removal was needed due to chronic biofilm in two cases, performing TAR after 6 months from the mesh explantation.

## Discussion

The first mandatory step before approaching these preoperative techniques is performing a volumetric CT scan to evaluate diameters and volumes of abdominal cavity and hernia sac, as well as to study the characteristics of lateral abdominal and rectus muscles (13). Tanaka et al. (9) was the first author to provide an objective method for calculating diameters and volumes according to CT findings. In fact, he defines LODH based on these radiologic measurements: a relation VIH/VAC over 20% would cause a traumatic reduction and possible postoperative complications. Furthermore, CT scan has been improved by more accurate radiologic images, which can obtain the length and thickness of the lateral muscles, especially in order to compare the effect of the BT (14).

Ibarra-Hurtado et al. (2) initially reported the administration of BT on the lateral abdominal wall muscles to obtain transient flaccid paralysis and decreasing the transverse diameter of the defect. The neurotoxin was demonstrated to enlarge the abdominal cavity by stretching and flattening of the retracted lateral abdominal wall. Likewise, BT optimizes the preoperative period of patients with LODH, reducing tension on the reconstructed abdominal wall and increasing the abdominal capacity prior to elective hernia repair (5). To assess its correct administration, we applied the combination of ultrasonographic and electromyographic guidance before every injection. Unlike other authors (15), our group relies on this association to check the efficiency of the administration, choosing the best infiltration point into the muscle, especially in cases of denervation or fibrosis. Therefore, this combined management would allow the modification of the injection point, improving its effectiveness (10).

In our study, significant changes in abdominal volumes and hernia transverse diameters in CT scan have been demonstrated, comparing before and after with BT and PPP combination. However, it may be difficult to try to measure and quantify the actual effect of each technique regardless of whether it was done in an objective way. Possibly, this benefit may be represented in the measurements found in the CT scan control, after its application. Although there was no significant reduction in the transverse diameter of the hernia defect, this fact, in addition to the length and width modifications in muscle retraction due to BT, probably contributed and helped the fascial free-tension closure.

Use of PPP in the treatment of LODH has been previously reported (6–8, 16–18), providing complete repair and reducing complications, such as the ACS and the restrictive respiratory disease. Tanaka et al. (9) administers a volume of air equivalent to the VIH observed in the preoperative CT scan, recommending PPP when the VIH/VAC ratio is more than 25%. On the other hand, Sabbagh et al. (8) uses the relation between VIH and total peritoneal content (PV). This group considers that PV is predictive of tension-free fascial closure in LODH and represents a more selective measurement of volume in the abdominal cavity: if the preoperative VIH/PV is more than 20%, the need for preoperative techniques such as PPP or BT can be predicted before hernia repair.

There is no consensus in the literature about the amount of air and duration of insufflation that must be administered (6, 17, 18). The main disadvantage is that the volumes reported are not based on an objective factor, and measurements are not completely feasible, so only the symptoms of the patient determine the volume to insufflate. Our group applies three times the amount of VIH (in cc), ~1 L of air per day to achieve respiratory accommodation toward the abdominal cavity. According to some authors, the most accurate way of knowing when to stop the air infusion is performing a CT scan after the procedure and calculating VIH and VAC (4, 7, 8). Anyway, further studies are needed to calculate the correct PPP volume to predict a successful fascial closure, avoiding the development of ACS.

Our group has previously reported the advantages of the combination of PPP and BT (3). So, based on the study of the hernia defect diameters and the VIH/VAC ratio, an algorithm was developed for the management of LODH, which has been modified according to our experience in this field. So, a primary fascial closure was achieved in 96.3% of the patients with LODH, just needing mesh bridging in three cases. A decrease of 15% of the VIH/VAC allowed the reintroduction of the hernia sac volume inside the abdominal cavity, improving respiratory adaptation after free-tension fascial closure. This conclusion would demonstrate the beneficial and synergic effect reached by the combination of both procedures, compared with the results obtained by each technique separately (4, 19).

This study was not performed without limitations. First, it is a retrospective observational descriptive study, with their drawbacks and disadvantages. According to our criteria, the follow-up period was not too long in the last patients of the series, although sufficient to be able to draw conclusions in our analysis; they completed 1-year controls, and no recurrence was detected during this period. Unfortunately, the effects of PPP and BT cannot be independently analyzed in this study. Furthermore, as we have previously described, the actual effect of BT has been difficult to demonstrate in an objective way, although important modifications of CT scan were observed. Thus, PPP would be considered more useful in the management of both volumes (directly on the VIH/VAC rate), independently of size defect, and BT administration would be more successful to reduce defect diameters, especially the hernia transverse diameter. Possibly, further studies about other ways of measurement of the preoperative use of BT and PPP are needed to confirm our results.

On the other hand, it is also important to remark the homogeneity of our outcomes because of the fact that all procedures were performed by five specialized hernia surgeons, decreasing mistakes due to lack of experience or modifications in the preoperative and surgical techniques, although the mesh election (PPL or PVDF) depended on the surgeon preferences.

As a conclusion, our arguments suggest that the combination of both techniques is useful in the management of LODH, to treat diameters and volumes of the hernia as a preamble to the surgical repair. PPP and BT are safe techniques with good patient tolerance. Although more prospective studies comparing both techniques are needed to confirm these results, BT and PPP significantly reduce the VIH/VAC ratio, which constitutes an important factor in the treatment of LODH.

## Data Availability Statement

The datasets generated for this study are available on request to the corresponding author.

## Ethics Statement

The studies involving human participants were reviewed and approved by Ethics Committee from La Fe Hospital. The patients/participants provided their written informed consent to participate in this study.

## Author Contributions

JB-L was the main author and creator of the idea of the paper. OC-S was the second author, reviewer, and took part in the discussion. AT-G was the author of the methods and the references reviewer. SP-S was the reviewer and translator of the paper.

### Conflict of Interest

The authors declare that the research was conducted in the absence of any commercial or financial relationships that could be construed as a potential conflict of interest.
